# Combination of *Oxalobacter Formigenes* and *Veillonella Parvula* in Gastrointestinal Microbiota Related to Bile-Acid Metabolism as a Biomarker for Hypertensive Nephropathy

**DOI:** 10.1155/2022/5999530

**Published:** 2022-05-17

**Authors:** Xin Li, Li Wang, Shaojun Ma, Shaohui Lin, Chunyan Wang, Haiya Wang

**Affiliations:** ^1^Department of General Medicine and Geriatrics, The Second Affiliated Hospital of School of Medicine, Linping Campus, Zhejiang University, No. 369, Yingbin Rd, Nanyuan St, Linping District, Hangzhou, Zhejiang Province 311100, China; ^2^Department of Geriatrics, Renji Hospital, School of Medicine, Shanghai Jiaotong University, No. 145, Shandong Rd., Shanghai 200001, China; ^3^Department of Geriatrics, Shanghai Ninth People's Hospital, School of Medicine, Shanghai Jiaotong University, No. 639, Zhizaoju Rd., Shanghai 200011, China; ^4^Shanghai Aikesuo Biomedical Technology L.L.C, Shanghai, China; ^5^Tomedex L.L.C, San Jose, CA, USA

## Abstract

The human microbiome is a mixed group of microorganisms, which individually consists of 10–100 trillion symbiotic microbial cells. The relationship between gastrointestinal microbiota and blood pressure has been verified and the intestinal microbiota of chronic kidney disease (CKD) patients in the distribution of bacterial species is different from the flora of people with no CKD. The purpose of this research is to study the different intestinal microbiota of hypertensive patients with and without nephropathy and to find possible biomarkers of hypertensive nephropathy (H-CKD). The subjects of this research were divided into three groups, healthy control group, hypertension group, and hypertensive nephropathy group. Sequencing, bioinformatics, and statistical analysis were performed on the 16S rRNA gene of the subjects' stool samples. This research study showed the differences of intestinal flora as biomarkers in hypertension patients with and without nephropathy; it investigated the relationship of the differences in the intestinal microbiota with bile-acid metabolism; it also explored bile-acid metabolism mechanism of intestinal microbiota differences in hypertension with or without nephropathy. In summary, the difference in the combination of *O. formigenes* and *V. parvula* in the gastrointestinal microbiota is related to bile-acid metabolism in hypertensive patients and can be one of the factors causing CKD. It is the first time to report such a biomarker or pathogenic factor of H-CKD in the world.

## 1. Introduction

Chronic kidney disease is an important contributor to morbidity and mortality in the world. It has been recognized as a risk factor for cardiovascular disease and a risk multiplier in patients with hypertension and diabetes [1, 2]. Despite major advances in the identification of key pathophysiological mechanisms and in treatment, hypertension remains one of the most important causes of acute and chronic cardiovascular disease [3, 4]. It is believed that the etiology of hypertension depends on the complex interplay of both genetic and environmental factors [5–7]. With sequencing technology development, more and more researchers switched their attention from dietary components to gastrointestinal microbiota (GM) [8–10].

The potential role of the GM in the altering health status of the hosts has drawn considerable attention. Some studies report that changes in the structure of the intestinal flora are directly related to hypertension, and the richness and diversity of the intestinal flora in hypertensive patients have decreased significantly, specifically as *Prevotella* and *Klebsiella* significant increase, while the content of beneficial bacteria decreases [11]. Intestinal flora-induced hypertension also affects host gene expression and basic metabolic processes [12]. It is generally believed that the intestinal flora produces metabolites, such as farnesoid X Receptor (FXR) signal antagonists, TGR5 signal agonists, *β*-taurocholic acid, and other primary bile acids [13, 14], thereby promoting atherosclerosis and calcification of blood vessel walls by affecting cholesterol metabolism to indirectly affecting the occurrence and development of hypertension. In addition to multiple factors, such as genes, inflammation, and neuroendocrine system, changes in the intestinal microbiota attract our attention as a new target for diagnosis and treatment of hypertension.

It is known that total bile acid (TBA) level of hypertensive patients is significantly higher than that of nonhypertensive patients. The TBA level is related to the grade of hypertension and has a certain correlation with structural damage to the kidney and the heart. TBA, a metabolite of cholesterol in the liver, provides a signal from the host to maintain the balance of the intestinal microbiota through hepatointestinal circulation [15, 16]. Researchers are currently focusing on bile salt hydrolase (BSH), which can enzymolyze the C-24-N-acyl bond of bile salt bound by glycine or taurine into free bile acid. Many bacteria, for example, *Bacteroides*, *Clostridium*, *Lactobacillus*, and *Bifidobacteria*, have BSH activity, which means the imbalance of intestinal flora probably influences the metabolism of bile acids [16]. Since TBA and GM are related to hypertension and kidney damage, and gut bacteria are involved in the metabolism of bile acid, we explore the relationship between GM and bile acid metabolites in patients with/without nephropathy to predict the incidence of the hypertension related kidney diseases in hypertension population.

## 2. Materials and Methods

### 2.1. Study Design and Sample Collection

All patient samples were newly diagnosed as primary hypertension or hypertension-related nephropathy at Renji Hospital, School of Medicine, Shanghai Jiaotong University. The diagnosis of hypertension is that the systolic blood pressure (SBP) is ≥140 mmHg and/or the diastolic blood pressure (DBP) is ≥90 mmHg after repeated examinations [17]. The patients who met the following two conditions are diagnosed with hypertension-related nephropathy: (1) Essential hypertension; (2) 30 ≤ eGFR ≤60 ml/min and/or urine albumin-creatinine ratio (ACR) ≥30 mg/g. Totally, we collected 51 individual stool and blood samples for this research, including 10 healthy controls (HC), 30 hypertensive patients with no nephropathy (hypertension), and 11 hypertensive nephropathy patients (H-CKD). The healthy individuals were selected from the volunteers to match disease groups of patients with age, gender, and body mass index (BMI). The exclusion criteria for the three groups are as follows: (1) Patients with a history of diabetes or fasting blood glucose higher than 110 mg/dL (6.11 mmol/L) or urinary albumin/creatinine >2.5; (2) patients with primary or secondary increasing urinary protein or kidney damage such as rheumatic connective tissue disease, acute and chronic glomerulonephritis, multiple myeloma et al; (3) patients with secondary hypertension such as protoaldehyde/adrenaline adenoma/renal artery stenosis/pituitary disease, etc.; (4) cerebrovascular diseases occur within 2 months, such as cerebral hemorrhage, cerebral infarction, transient ischemic attack, subarachnoid hemorrhage, angina or myocardial infarction, PTCA, CABG, persistent atrial fibrillation and atrial flutter, combined with stenotic arteriosclerosis etc.; (5) patients with a history of congenital and rheumatic heart disease, and cardiac insufficiency (NYHA cardiac function class III or higher); (6) patients with a history of acute infection, acute bleeding or blood transfusion within 2 months, and taking any antibiotic and microbial preparation within 1 month; and (7) patients with severe liver and kidney dysfunction (eGFR less than 30), malignant tumors, inflammatory bowel disease, and acute and chronic pancreatitis. This study was approved by the Ethics Committee of Renji Hospital, School of Medicine, Shanghai Jiaotong University, and all the participants in the study signed an informed consent.

### 2.2. Experiment and Analysis of Serum Bile Acids Components

After 8-hour of fasting, 2 ml of venous blood was drawn from each participant for the bile acids test. We performed liquid chromatography-tandem mass spectrometry (LC/MS) to analyze bile acid components in serum samples. First, we pipetted 50 *μ*l serum into 1 ml EP tube with 10ul 2.5 *μ*g/ml internal standard mixture bile-acid solution and centrifuged with the speed of 15,000 × g for 10min at 4°C. Then, we transferred 100 *μ*l of the supernatant into 100 *μ*l pure water and mix. Finally, we pipetted 10 *μ*l of mixture for LC-MS/MS analysis.

We used the AB5600 Triple TOF system(AB SCIEX) to identify and quantify the bile acids component. The different bile acids were separated by an A2.1 × 100 mm XBridge Peptide BEH C18 column with a 4 × 2 mm guard column. We also used a controlled gradient of mobile phase A (0.1% formic acid and 10 mM acetic acid amine in water), and mobile phase B (0.1% formic acid in 80% methanol and 20% acetonitrile) to separate the components at 40°C, a flow rate of 0.4 ml/min. We set Triple TOF as follows: ion spray voltage floating was in negative mode −4500 V; curtain gas was 30; ion source gas 1 and 2 were 50; source temperature was 550°C. Under automatic MS/MS acquisition, we set the m/z range 200–800 Da to acquire TOF MS scans and the m/z range 50–800 Da to product ion scan. The accumulation time of TOF MS scans and product ion scans was fixed at 19.993 min. The collision energy of the product ion scan was −45 V ± 20 V, and the declustering potential was −80 V.

### 2.3. DNA Extraction and 16S rRNA Gene Sequencing

We performed 16S rRNA gene sequencing. Briefly, after extracting bacterial genomic DNA by the E.Z.N.ATM Mag-Bind Soil DNA Kit (OMEGA, Switzerland), the 16S rRNA gene V3-V4 region was amplified by polymerase chain reaction (PCR) (PrimerF: CCTACGGGNGGCWGCAG; PrimerR: GACTACHVGGGTATCTAATCC) and sequenced using the MiSeq platform (Illumina, San Diego, California, USA).

### 2.4. Bioinformatic Analysis of 16s rRNA Gene Sequencing

The 16S rRNA gene sequencing data were analyzed by quantitative insights into microbial ecology (QIIME1) [18]. Then, we used the Vsearch plugin to cluster sequences into operational taxonomic units (OTUs) at 97% identity and the taxonomy was assigned against the Greengenes database (V.13.8). After filtering, each sample had an average of 72473 reads of alpha value, using q2 diversity to perform *β* diversity analysis at 2500 rare sampling depths. The metagenomes of the gut microbiome were imputed from 16S rRNA gene sequences by PICRUSt (Phylogenetic Investigation of Communities by Reconstruction of Unobserved States) [19].

### 2.5. Statistical Analysis

We applied the method of linear discriminant analysis of effect (LEfSe) to determine the abundance of taxa or pathway differences between cases and control [20]. This method first used the nonparametric factorial Kruskal–Wallis sum-rank test to detect features with significant differential abundance and then used linear discriminant analysis (LDA) to calculate the effect size of each feature.

The relationship between the microbiota and bile acid profile was analyzed by canonical correspondence analysis/redundancy analysis (CCA/RDA) [21]. The R heatmap package was used to analyze whether the microbiome is significantly correlated with bile acid and to calculate the Spearman coefficient [22]. The function of the microbiome was analyzed by the Kyoto Encyclopedia of Genes and Genomes (KEEG) [23], and the predictive function was analyzed by the ANOVA test to see if there were significant differences among the groups.

Statistical analysis was performed with R (V3.51). The relative taxa abundance was arcsine square root transformed before regression tests (glm function). We used multivariate stepwise logistic regression analysis and the caret package to identify the genera that best distinguished hypertensive patients with or without nephropathy from controls. Other statistical analysis tools were Fisher's exact test, the Kruskal–Wallis test, and partial Spearman's rank correlation (PResiduals package).

## 3. Results

### 3.1. GM Diversity in Hypertension with or without Nephropathy

In order to determine whether changes in gut microbes are related to H-CKD, we studied the fecal microbiota in a well-characterized cohort. All participants provided stool and blood samples under their approval for 16S rRNA gene sequencing and analysis, and bile-acid test. There are no significant differences in other characteristics, except for a family history of early-onset cardiovascular and cerebrovascular diseases: Hypertension vs HC (*p*=0.00136); H-CKD vs Hypertension (*p*=0.00106), by the Kruskal–Wallis test. The detailed demographic, clinical, and hypertensive features of the cohort are in Table 1.

**Figure 1:**
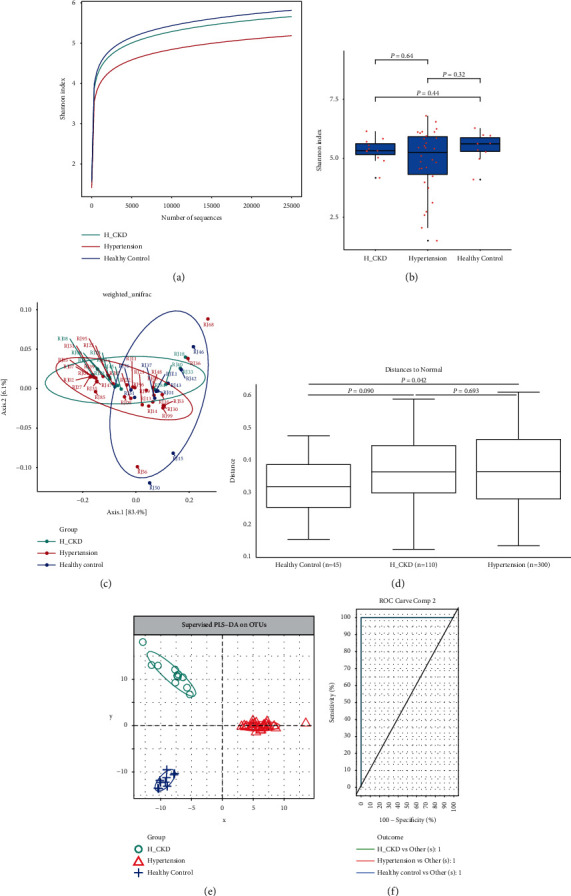
Comparisons of alpha-diversity and beta-diversity among the groups of H-CKD, hypertension, and healthy control. (a) Rarefaction curves for the mean of Shannon index in the three groups. The curve for each group is nearly smooth when the sequencing data are abundant enough with few new OTUs detected; (b) comparison of diversity in the three groups accessed by the Shannon index. Compared with the control group tested by the Kruskal–Wallis test, the *α* diversity of H-CKD group and hypertensive groups do not significantly decrease at the genus level; (c) PCoA analysis based on the weighted UniFrac matrix, the two components were 6.1% and 83.4%, respectively; (d) comparison of beta-diversity among the three groups. Overall, the fecal microbiota compositions do not significantly differ among the three groups, while the difference between Hypertension and healthy control is significant; (e) PLS-DA shows significant differences among the three groups; and (f) ROC analysis for the predictive value. The AUC of all the three groups are 1. OTU, operational taxonomic units; PCoA, principal coordinate analysis; PLS-DA, partial least squares discrimination analysis.

In this cohort study, rarefaction was performed to estimate the total number of operational taxonomic units (OTUs) of the stool samples; the sequencing data were abundant enough that few new OTUs were found. Based on the genera profile, the Shannon index was calculated to estimate the within-sample *α* diversity. The rate of acquisition in the healthy group exceeded the one of new OTUs acquisition in the hypertensive groups, which indicated that the bacterial diversity level of the hypertensive groups was lower (Figure 1(a)). Compared with the control group by the Kruskal–Wallis test, the *α* diversity of H-CKD and hypertension groups did not significantly decrease at the genus level (H-CKD vs hypertension, *p*=0.64; H-CKD vs HC, *p*=0.44; hypertension vs HC, *p*=0.32 (Figure 1(b)). To evaluate the overall diversity of microbial composition, we performed a principal coordinate analysis (PCoA) based on unweighted UniFrac, weighted UniFrac, Bray–Curtis distance, and partial least square discriminant analysis (PLS-DA). The multivariate permutation test (MPT) showed that there were no significant differences in the composition of the intestinal taxonomy between the H-CKD group and other groups, while significant differences were found between hypertension and HC as two main coordinates PC1 and PC2 (unweighted UniFrac: 5.6% and 9.2%; weighted UniFrac: 6.1% and 83.4%; Bray–Curtis: 7% and 17.1%; H-CKD vs hypertension, *p*=0.693; H-CKD vs HC, *p*=0.090; hypertension vs HC, *p*=0.042) (Figures 1(c) and 1(d); Supplement Figure 2). The results of PLS-DA showed that the difference and the distinction were significant among the three groups, ignoring the random error (Figure 1(e)), suggesting that the prediction model was good enough and intestinal microbiota could be used as a strong risk because all the AUCs were 1 (Figure 1(f)). There was no significant difference in *α* diversity of intestinal flora between antihypertensive and non-antihypertensive medicine in the H-CKD group (Supplement Figure 1C), as well as in the group of hypertension without CDK (Supplement Figure 1D).

### 3.2. Bacteria Differential Abundance in Hypertension and H-CKD versus HC

**Figure 2:**
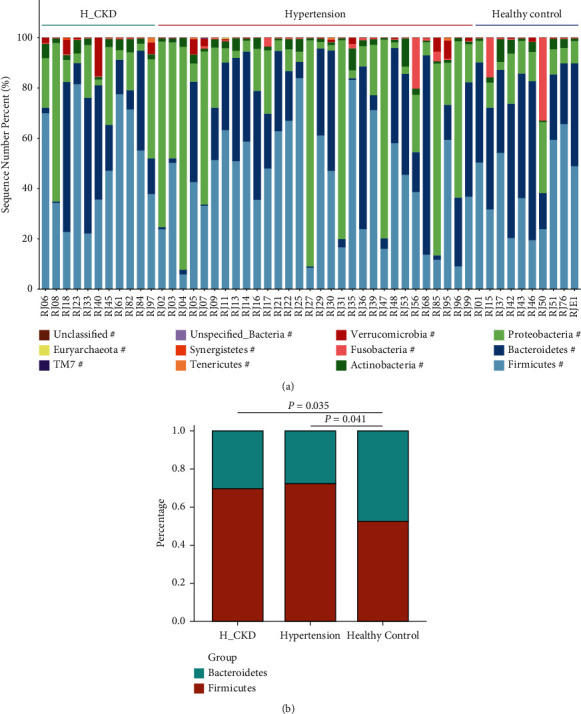
Variations of fecal microbiota in H-CKD, hypertension and healthy control. (a) Relative proportions of bacterial phyla in H-CKD (*n* = 11), hypertension (*n* = 30), and healthy control (*n* = 10); different colors represent different phyla; It suggests biodiversity in every individual; no significant different phylum was found among the three groups (#: *p* > 0.05); (b) rate of relative proportions of F/B. F/B in both H-CKD and hypertensive is significantly lower than it in Healthy Control group. F/B: *Firmicutes/Bacteroidetes.*

**Figure 3:**
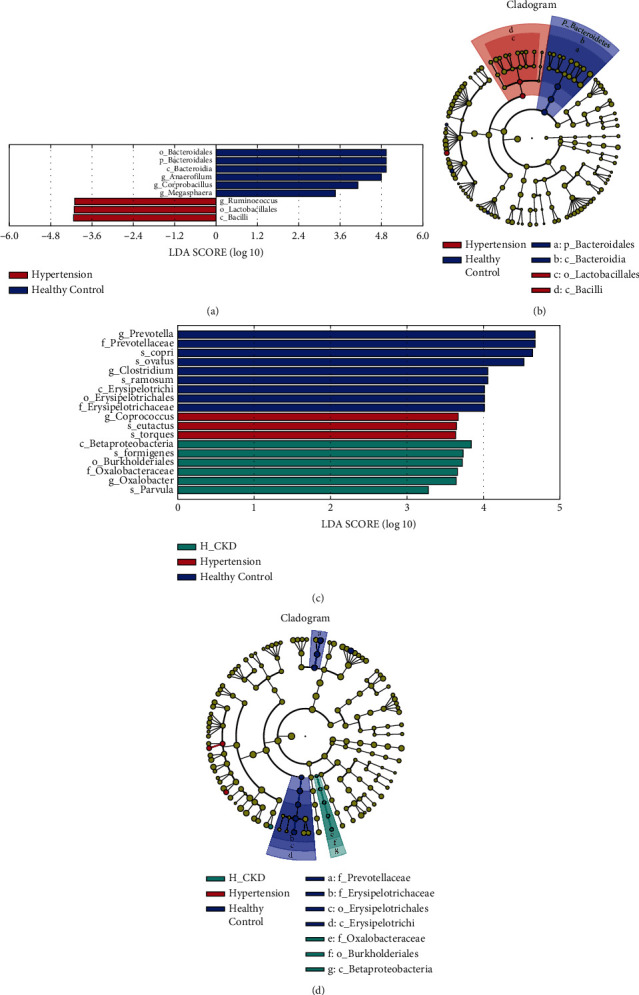
Characteristics of Gut Microbiota in the HC, hypertension and H-CKD. (a) Diagram of the LDA score at genus level between hypertension and HC; the enriched genus in hypertension is red, and blue in HC; (b) cladogram at genus level shows the original bacterial tree form the central point, the circles form inside to outside representing phylum to genus; The *c-Bacteroidia* is the main enriched in HC, and the *c-Bacilli* is the main enriched in hypertension; (c) diagram of the LDA score at genus levels among hypertension, H-CKD, and HC; the enriched genus is green in H-CKD, red in hypertension, and blue in HC; (d) cladogram at genus level shows the original bacterial tree form the central point, the circles form the inside to outside representing phylum to genus; the *c-Erysirelattishi* is the main enriched in HC, and the *c-Betaproteobacteria* is the main enriched in H-CKD. phylum to species: (p) phylum; (c) class; (o) order; (f) family; (g) genus; and (s) species.

To identify differential abundant taxa, we used LEfSe to analyze the differences in fecal Microbiota composition. *Bacteroidetes*, *Firmicutes*, and *Proteobacteria* were the most dominant phylum in all three groups (Figure 2(a)). The ratio of *Firmicutes*/*Bacteroidetes* (F/B) in both the H-CKD and hypertension groups were significantly lower compared to the HC group (H-CKD, F/B = 2.30; hypertension, F/B = 2.60; HC, F/B = 0.90. H-CKD vs HC, *p*=0.035; hypertension vs HC, *p*=0.041), analyzed by the Kruskal–Wallis test (Figure 2(b)). There was no significant difference of F/B between the hypertension group and the H-CKD group. LefSe analysis revealed that compared with the HC group *Anerofilum* (*p*=0.002, LDA score = 4.8) and the *Coprobacillus* (*p*=0.003, LDA score = 4.1) were significantly downexpressed and *Ruminococcus* (*p*=0.006, LDA score = −4.2) was significantly upexpressed in hypertension with/without CKD patients (*p* < 0.05, LDA score >2.0) (Figures 3(a) and 3(b)). After comparing the bacterial taxa among the three groups, we found 19 bacterial taxa of distinctly relative abundances. Compared with the hypertension group, the abundances of *Veillonella parvula* (*V. parvula*) (*p*=0.017, LDA score = 3.28) and *Oxalobacter formigenes* (*O. formigenes*) (*p*=0.019, LDA score = 3.73) in the H-CKD group were significantly upexpressed, and the contents of *Coprococcus eutactus* (*p*=0.006, LDA score = 3.65) and *Ruminocccus torques* (*p*=0.042, LDA score = 3.63) were downexpressed. Meanwhile, the abundances of *Bacteroides ovatus* (*p*=0.047, LDA score = 4.52), *Clostridium ramsum* (*p*=0.015, LDA score = 4.06), *Prevotella copri* (*P. copri*) (*p*=0.018, LDA score = 4.68), and class *Erysipelotrichi* (*p*=0.029, LDA score = 4.01) were found as the feature of gut bacteria in the healthy group by LEfSe analysis, all the four bacteria were downexpressed in both the hypertension group and H-CKD group (Figures 3(c) and 3(d)). The different biological abundance of the taxa among the three groups is shown in Supplement Figure 3.

**Figure 4:**
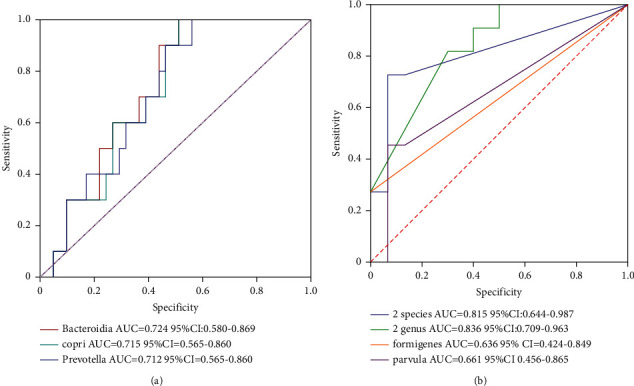
ROC analysis for the predictive value. (a) Relative abundance of 3 hypertension-associated genera that distinguish hypertension from controls is plotted on a logarithmic scale, and the values of zero are assigned and (b) relative abundance of 2 H-CKD-associated genus and 2 species that distinguish H-CKD from controls are plotted on a logarithmic scale, and the values of zero are assigned.

To investigate the different composition of bacteria in the gut microbiota between hypertensive patients and healthy individuals, we separated 51 volunteers into two groups: a healthy control group and a hypertensive group, including hypertension with and without nephropathy patients. By LEfSe Analysis, we found that in the hypertensive group, the abundance of *Anaerobes* and *Parabacterium* decreased, while the abundance of *Ruminococcus* increased (Figures 3(c) and 3(d)). Then, we assessed the potential values of the gut microbiota as biomarkers. First, we tested the potential biomarker of hypertension using *Bacteroidia* as predictor to generate an area under receiving operating characteristics curves (AUROC) of 0.724 (95% CI 0.580 to 0.769); using *Prevotella* as predictor to generate AUROC of 0.712 (95% CI 0.565 to 0.860); using *P. copri* as a predictor to generate AUROC of 0.715 (95% CI 0.565 to 0.860) (Figure 4(a)). Next, we tested the potential biomarkers of H-CKD in the hypertensive group. The respective AUROC generated by *O. formigenes* and *V. parvula* were 0.636 (95% CI 0.424 to 0.849) and 0.665 (95% CI 0.456 to 0.865) (Figure 4(b)). In addition, multivariate stepwise logistic regression analysis was applied to the list of genera and species related to H-CKD to identify the taxa, which best distinguish H-CKD from hypertension. The results indicated that the combination of *Oxalobacter* and *Lachnospiraceae Clostridium* could be a biomarker to predict H-CKD from simple hypertension with the AUC of 0.836 (95% CI 0.709 to 0.963). We also found that the combination of *O. formigenes* and *V. parvula* could distinguish H-CKD from hypertension with an AUC of 0.815 (95% CI 0.644 to 0.987) (Figure 4(b)).

### 3.3. Correlations between the Gut Microbiota and Bile Acid Metabolism

**Figure 5:**
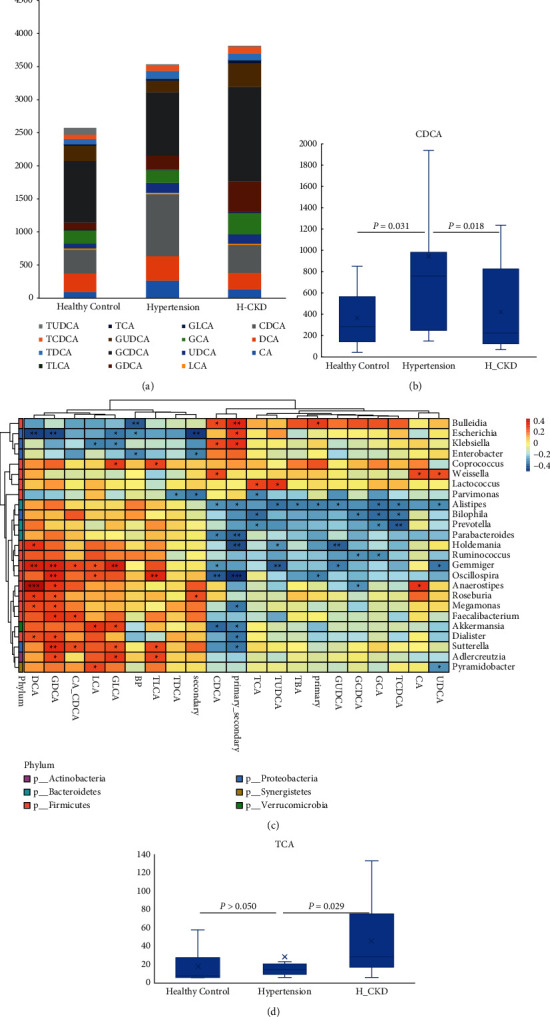
Relationship between gut microbiota and bile acid metabolism in HC, H-CKD, and hypertension. (a) Different contents of bile acid metabolism among HC, H-CKD and hypertension groups and (b) concentration of CDCA is significantly higher in both H-CKD and hypertension than in HC. The content of CDCA in hypertension is higher than that in H-CKD (HC vs hypertension, *p*=0.031; hypertension vs H-CKD, *p*=0.018), by the Mann–Whitney *U* test; (c) heatmap shows that the partial Spearman correlation coefficients between genera and bile acid metabolism (^*∗*^: 0.01 < *p* ≤ 0.05, ^*∗∗*^: 0.01 < *p* ≤ 0.01, ^*∗∗∗*^: *p* ≤ 0.001); and (d) concentration of TCA of the H-CKD group is significantly higher than that of the hypertension group and has no difference between hypertension and HC. CA: cholic acid; DCA: deoxycholic acid; CDCA: chenodeoxycholic acid; LCA: lithocholic acid; UDCA: ursodeoxycholic acid; GCA: glycocholic acid; GLCA: glycolithocholic acid; GDCA: glycodeoxycholic acid; GCDCA: glycochenodeoxycholic acid; GUDCA: glycoursodeoxycholic acid; TCA: taurocholic acid; TLCA: taurolithocholic acid; TDCA: taurodeoxycholic acid; TCDCA: taurochenodeoxycholic acid; TUDCA: tauroursodeoxycholic acid.

Correlation of clinical variables with disease-associated bacteria showed that the mean levels of different types of bile acid were different in H-CKD, hypertension, and HC (Figure 5(a)). Although the level of total bile acid did not change significantly, the content of chenodeoxycholic acid (CDCA) was significantly higher in both H-CKD and hypertension than in HC. The content of CDCA in hypertension was higher than that in H-CKD (HC vs hypertension, *p*=0.031; hypertension vs H-CKD, *p*=0.018), by the Mann–Whitney *U* test (Figure 5(b)). The content of taurocholic acid in H-CKD was significantly higher than that in the hypertension group. However, there was no significant difference between hypertension and HC (HC vs hypertension, *p* > 0.050; hypertension vs H-CKD, *p*=0.029), by the Mann–Whitney *U* test (Figures 5(c) and 5(d)). There was no significant difference of bile acid metabolism between antihypertensive and nonantihypertensive medicines in the H-CKD group (Supplement Figure 1A), as well as in the group of hypertension with no CKD (Supplement Figure 1B).

**Figure 6:**
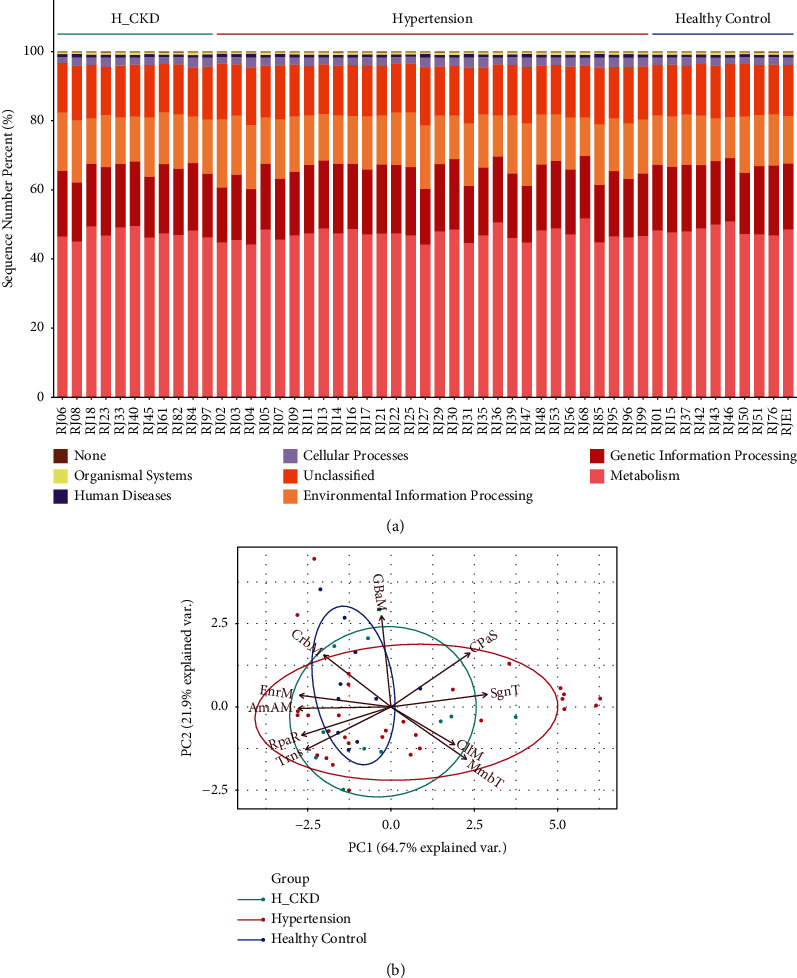
Predicted metagenomes functional analysis by PICRUSt. (a) Bar chart shows that different relative abundance predicted function of gut bacteria in all individuals on the first KEEG pathway level and (b) predicted functions on the second KEEG pathway level. PC1 represents the first principal component, and the percentage represents the contribution of the first principal component to the sample difference; PC2 represents the second principal component, and the percentage represents the contribution of the second principal component to the sample difference (PC1:64.7%; PC2: 21.9%). The direction and length of the arrow represent the degree of dominance of the prediction functions in the sample/group in that direction, the longer the length, the stronger the dominance. CPaS: cellular processes and signaling; Trns: translation; EnrM: energy metabolism; RpaR: replication and repair; AmAM: amino acid metabolism; CrbM: carbohydrate metabolism; MmpT: membrane transport; SgnT: signal transduction; MmbT: membrane transport; CllM: cell motility GBaM: glycan biosynthesis and metabolism; EnrM: energy metabolism.

In addition, using partial Spearman's rank-based correlation test, we investigated whether the difference of bile acid profile is related to intestinal bacteria. It was found that deoxycholic acid is positively correlated with *Anaerostipes* (*R* = 0.46, *p* < 0.01), *Firmicutes* (*R* = 0.37, *p* < 0.05), and *Clostridium* (*R* = 0.38, *p* < 0.05), while it is negatively correlated with *Escherichia* (*R* = −0.43, *p* < 0.01), *Enterobacteriaceae* (*R* = 0.48, *p* < 0.05), and *Proteobacteria* (*R* = 0.47, *p* < 0.05). Glycodeoxycholic acid is negatively correlated with *Escherichia* (*R* = −0.41, *p* < 0.05), while it is positively correlated with *Oscillospira* (*R* = 0.37, *p* < 0.05). *Oscillospira* is positively related to taurolithocholic acid (*R* = 0.38, *p* < 0.05) and negatively related to CDCA (*R* = −0.37, *p* < 0.05). The results show a negative correlation between *Prevotella* and taurochenodeoxycholic acid (*R* = −0.37, *p* < 0.01), and a positive correlation between *Coprococcus* and glycolithocholic acid (*R* = 0.32, *p* < 0.05) (Figure 5(c)). We analyzed the functional potential of the gut microbiome by PIRUST to investigate the functional changes of the microbial among the H-CKD, hypertension, and HC groups. Metabolism, genetic, and environmental information processing are the main functions of the human gastrointestinal microbiota. There were no differences of these main functions among the H-CKD, hypertension, and HC groups (Figure 6(a)). Using principal coordinate analysis, we found that the dominance functions of membrane transport, cell motility, signal transduction, cellular processes, and signaling in H-CKD and hypertension groups were stronger than those in the healthy control group (Figure 6(b)). We also observed that D-glutamine (ANOVA test, *p*=0.014) and its metabolism and glycosphingolipid biosynthesis-lacto and neolacto series (Anova test, *p*=0.014) were significantly different between the Hypertension and HC groups.

## 4. Discussion

In this research, we studied the structure of the fecal microbiome of hypertension with or without nephropathy by 16S rRNA gene sequencing and analysis. The results show that although there is no significant difference between healthy participants and hypertension with or without nephropathy, the microbial dysbiosis in the disease groups is characterized by changes in the relative abundance of the 19 bacterial taxa. The CDCA level of hypertensive patients without nephropathy is higher than that of individuals in the other two groups. It is probably related to the bile salt metabolism of *Bacteroides* and *Clostridium*.

All patients in the hypertension and H-CKD groups were newly diagnosed with hypertension or a hypertension-related kidney disease. Although multiple studies [8, 11, 24]discover lower species richness and evenness in the hypertensive group, our findings suggest that there is no difference in GM diversity between hypertensive patients with or without nephropathy and healthy controls. It is probably related to the grade of hypertension [25]. Since there are only 51 subjects in our study, including 10 healthy controls, 30 hypertension patients, and 11 hypertensive nephropathy patients, we are unable to sub-divide them by grade of hypertension. However, we found that the F/B of the disease group was significantly higher than the F/B of the healthy group, and *Bacteroides* was the dominant phylum of the healthy group, which was consistent with previous animal studies [26]. This suggests that the composition of the intestinal microbiome is different in patients with simple hypertension or hypertensive nephropathy. This suggests that hypertensive patients with and without nephropathy have different gut microbiota compositions.

The most notable findings of this research are as follows: (1) the intestinal characteristic bacteria of hypertension are *Coprococcus eutactus* and *Ruminococcus torques*; (2) the gut characteristic bacteria of hypertension-related nephropathy are *V. parvula* and *O. formigenes*; (3) the abundances of *Bacteroides ovatus*, *Clostridium Ramsum*, *P. copri*, and *Erysipelothrix spp.* of the hypertension and H-CKD groups are lower than healthy controls; and (4) the combination of *O. formigenes* and *V. parvula* can be a biomarker for H-CKD. Specifically, the combination of these two bacteria could better differentiate patients with hypertensive nephropathy from those with isolated hypertension.

After the regression analysis, *Coprococcus eutactus* and *Ruminococcus torques* could not be used as biomarkers to distinguish hypertension from HC. The accuracy of prediction relies on the number of subjects. *P. copri*, *Bacteroidia*, and *Prevotella*, which are the characteristic bacteria in the HC group, have good differentiation respectively. Previous studies [27] have proved that *Coprococcus spp.* has a high affinity for carbohydrates and can decompose carbohydrates to produce large amounts of butyric acid and acetic acid, also known as Short-Chain Fatty Acids (SCFAs). SCFAs are ligands for many G protein-coupled receptors (such as Gpr41, Gpr43, and Gpr109a) and olfactory receptor 78 (Olfr78) mainly found in the renal afferent arteriole and smooth muscle cells of the peripheral vascular system [28–30]. Renal afferent arterioles are the main place for storage and secretion of renin, which can increase blood pressure through the renin-angiotensin-aldosterone pathway. The studies [31] have shown that the combination of SCFA and Gpr41 can decrease blood pressure, while the combination of Olfr78 can increase the secretion of renin to raise blood pressure. This physiological opposition may be related to the different ligand affinities of these two receptors. High concentrations of SCFAs may activate Olfr78 to increase blood pressure. Therefore, it is speculated that *Coprococcus eutactus* can produce more SCFAs from carbohydrates, exceeding the upper limit to promote the development of hypertension.

We also found that the abundance of *P. copri* was significantly lower in hypertension and H-CKD groups. It could be affected by human diet and lifestyle. The previous research study [32] suggests that *P. copri* in the intestine of people often taking in a large amount of high-fiber diet has a stronger ability to decompose carbohydrates. However, Natarajan et al. [31] found that the abundance of *P. copri* in the hypertensive group was higher than that in the healthy controls. There is controversy regarding the beneficial or harmful effects of *P. copri* strain due to different diet patterns screened different *P. copri* strains, thus further research studies are required to investigate the relationship between *P. copri* and hypertension.

Our study focuses on the biomarkers of H-CKD. The combination of *O*. *formigenes* and *V*. *parvula* has a higher predictive value than other bacteria. In the hypertensive nephropathy group, *O*. *formigenes* is able to decompose oxalic acid to produce formic acid [33]. In addition, our study found that *V*. *parvula* was the dominant bacterium in the patients with hypertensive nephropathy. *V*. *parvula*, a Gram-positive bacterium with lipopolysaccharide (LPS), is an opportunistic pathogen that usually parasitizes the human gastrointestinal tract and oral cavity [34]. However, the specificity of *V*. *parvula* is insufficient because it is also found in autoimmune hepatitis [35]. Although, the mechanism of *O*. *formigenes* and *V*. *parvula* induce hypertensive nephropathy is unclear, for the first time, we found difference in the combination of these two bacteria in the hypertensive nephropathy group compared to the simple hypertension and HC people. The mechanism needs to be further studied. The characteristic bacteria in hypertensive nephropathy probably are different from those in other renal diseases. A study on IgA nephropathy (IgAN) shows that the significantly increased genera in the IgAN group are *Escherichia-Shigella*, *Hungatella,* and *Eggerthella*, which possess a pathogenic potential [36, 37]. It is also found that two genera in the *Lachnospiraceae* family are associated positively with renal function and negatively with phenylacetylglutamine metabolite [38]. Based on the correlation analysis of the influence of bile acid as an environmental factor on the intestinal flora, the result showed that Taurochenodeoxycholic acid (TCDCA) was negatively correlated with *Prevotella spp*. In association with the hepatic and intestinal circulation mechanisms of bile acids, it was speculated that the increase in venous plasma TCDCA may inhibit the growth of *Prevotella spp.*, therefore increasing blood pressure. However, there was no difference in TCDCA levels between the hypertension group and the healthy group. *Bacteroides* and a variety of bacteria are involved in the synthesis degradation CDCA, the effect of CDCA on blood pressure is still controversial [39]. Studies [40] shows that CDCA can act as an antagonist of the farnesoid X receptor to reduce blood pressure and regulate vascular tenses. This study also found that *Bacteroides* expression decreased and F/B increased in the hypertension group, which was consistent with previous research studies. *Bacteroides* expression is probably one of the pathogeneses of hypertension [41, 42]. By the correlation analysis, we found that CDCA levels were negatively correlated with *Oscillatoria spp.*, a Gram-positive anaerobic bacterium. Studies show that the level of *Oscillatoria spp.* is related to vegetarian diet patterns [43]. Hence, we speculated that a vegetarian diet might be beneficial to blood pressure control [44]. However, we did not find the difference in *Oscillatoria spp.* among these three groups by LEfSe analysis.

There was no statistical correlation found among hypertension, H-CKD, and HC groups in terms of DNA replication, repair, and certain amino acid metabolism. The PIRUST analysis, however, showed D-glutamine and D-glutamate metabolism, and glycosphingolipid biosynthesis-lacto and neolacto series were significantly different (*p*=0.014). Mels et al. [45] have found that the metabolism of D-glutamine and D-glutamate is one of the metabolic pathways of hypertension. Oxidative inflammatory damage of the vascular endothelium is one of the pathogeneses of hypertension. Glutamate is an important substrate of glutathione which is an effective antioxidant in the human body. Therefore, it is speculated that the level of glutamate affects the level of glutathione that mediates the oxidative inflammation of the vessel wall and further participates in the occurrence of hypertension. Other researchers [46] have found that the synthesis of neutral glycosphingolipids in the brain of stress-induced hypertension rats is significantly inhibited, and the content of glycosphingolipids is significantly negatively correlated with the prognosis of acute stroke. This suggests that glycosphingolipid biosynthesis-lacto and neolacto series are related to the compilations of hypertension.

The main advantages of our research study include collecting hypertensive nephropathy samples before renal protective interventions, using a cohort that includes any other diseases, discovering characteristic intestinal bacteria and preliminary discussion about the correlation between bile acid metabolism and GM in hypertension patients with/without nephropathy. Nevertheless, several limitations need to be noted. First of all, all the samples were collected at the local hospital. The number of the samples in this cohort is not large enough, so our research study is not universal but representative. Second, this research provides the insights for preventing hypertension with/without nephropathy and also increases the possibility of using the biomarker to diagnose hypertensive nephropathy. It provides evidence of association rather than causation.

## 5. Conclusion

The overall composition of the intestinal microbes has no significant difference in diversity, compared with hypertension and healthy controls. F/B increases significantly in hypertensive patients.

The intestinal characteristic flora of hypertension with no nephropathy are *Coprococcus eutactus* and *Ruminococcus torques*; the flora of hypertension with nephropathy are *V. parvula* and *O. formigenes*; and those of the healthy control are *Bacteroides ovatus*, *Clostridium ramsum*, *P. copri*, and *Erysipelothrix spp.* The combination of *O. formigenes and V. parvula* can be used as a biomarker for hypertension nephropathy.

The CDCA level of hypertensive patients without nephropathy is higher than that of healthy controls and that of hypertensive nephropathy patients. It is probably related to the bile-salt hydrolysis of *Bacteroides* and *Clostridium.*

## Figures and Tables

**Table 1:** Characteristics information of experimental groups.

Characteristics	Normal *n* = 10	HBP *n* = 30	H-CKD *n* = 11	*p* Normal vs HBP	*p* HBP vs H-CKD
Demographics					
Age, years median (Q1–Q3)	45 (42.0–51.5)	52.0 (40.0–65.3)	55.0 (43.5–64.5)	0.109	0.116
Gender, female, *n*(%)	5 (50.0%)	14 (46.7%)	5 (45.5%)	0.067	0.062
BMI, kg/m^2^, median, (Q1–Q3)	22.3 (21.0–24.3)	24.1 (22.9–25.8)	23.1 (22.4–24.8)	0.055	0.054
Behavior					
Smoke, yes, *n*(%)	1 (10.0%)	12 (40.0%)	5 (45.6%)	0.05	0.055
Alcohol, yes, *n*(%)	2(20.0%)	10 (33.3%)	4 (36.4%)	0.074	0.084
Hypertension					
History, yes, *n*(%)	3 (33.3%)	23 (76.7%)	8 (63.9%)	0.001	0.001
H grade-1, *n*(%)	—	4 (13.3%)	1 (9.0%)	—	—
H grade-2, *n*(%)	—	6 (20.0%)	6 (54.5%)	—	—
H grade-3, *n*(%)	—	6 (20.0%)	4 (39.1%)	—	—
Drug, no, *n*(%)	—	9 (30.0%)	6 (54.5%)	—	0.032
Time, *n*(%)					
0–5 years	—	22 (73.3%)	7 (63.6%)	—	0.010
5–10 years	—	6 (20.0%)	3 (27.2%)	—	—
>10 years	—	2 (6.7%)	1 (9.1%)	—	—

BMI: body mass index; History: family history of early-onset cardiovascular and cerebrovascular disease; H grade: hypertensive grade; drug: Five categories of commonly used antihypertensive drugs; Time: time of hypertension or H-CKD.

## Data Availability

The FASTQ files of raw data have been uploaded to NCBI and the Accession Number is PRJNA803617. By the information from NCBI, the data will be available when the article is published.

## Supplementary Materials

Supplementary Materials Supplement Figure 1: The Comparison of Bile Acids and Gut Microbiome Composition in Patients with and without Taking Antihypertensive medicines. Supplement Figure 2: PCoA Analysis of Gut Bacteria among HC, H-CKD, Hypertension. Supplement Figure 3: Normalized Abundance of Feature Bacteria in H-CKD, Hypertension and HC.
