# Supplementary material for: Combination of Oxalobacter Formigenes and Veillonella Parvula in Gastrointestinal Microbiota Related to Bile-Acid Metabolism as a Biomarker for Hypertensive Nephropathy
Source: Int J Hypertens. 2022 May 17;2022:5999530. doi: 10.1155/2022/5999530 (PMC9129936; doi:10.1155/2022/5999530)
Supplement: Supplementary Materials — Supplement Figure 1: The Comparison of Bile Acids and Gut Microbiome Composition in Patients with and without Taking Antihypertensive medicines. Supplement Figure 2: PCoA Analysis of Gut Bacteria among HC, H-CKD, Hypertension. Supplement Figure 3: Normalized Abundance of Feature Bacteria in H-CKD, Hypertension and HC. [file 5999530.f1.pdf]

Supplementary Materials

- **Supplement Figure 1**

Supplement Figure 1A

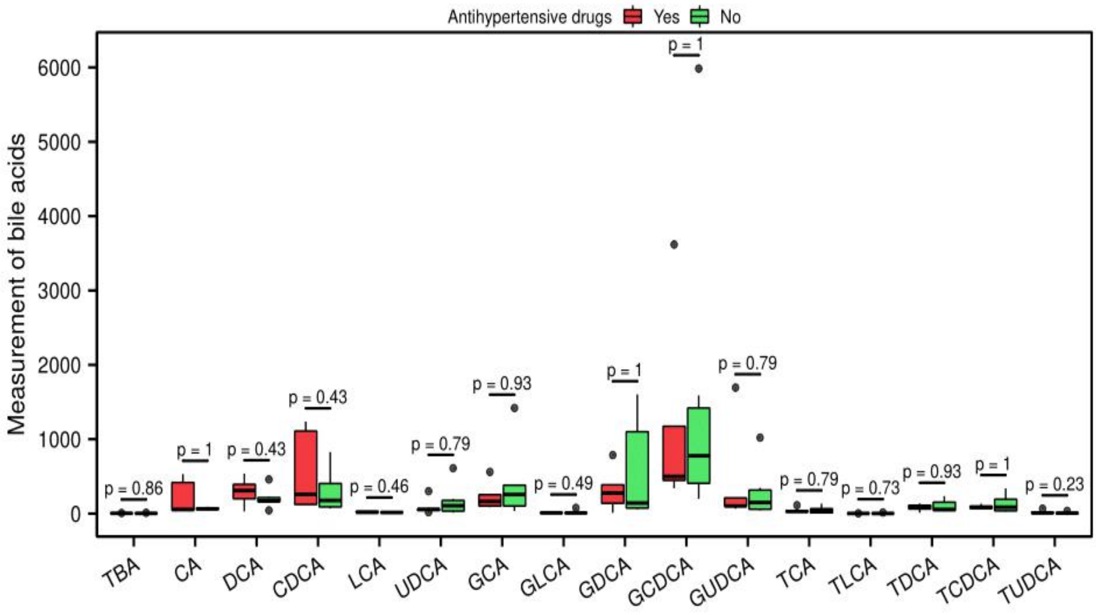

Supplement Figure 1B

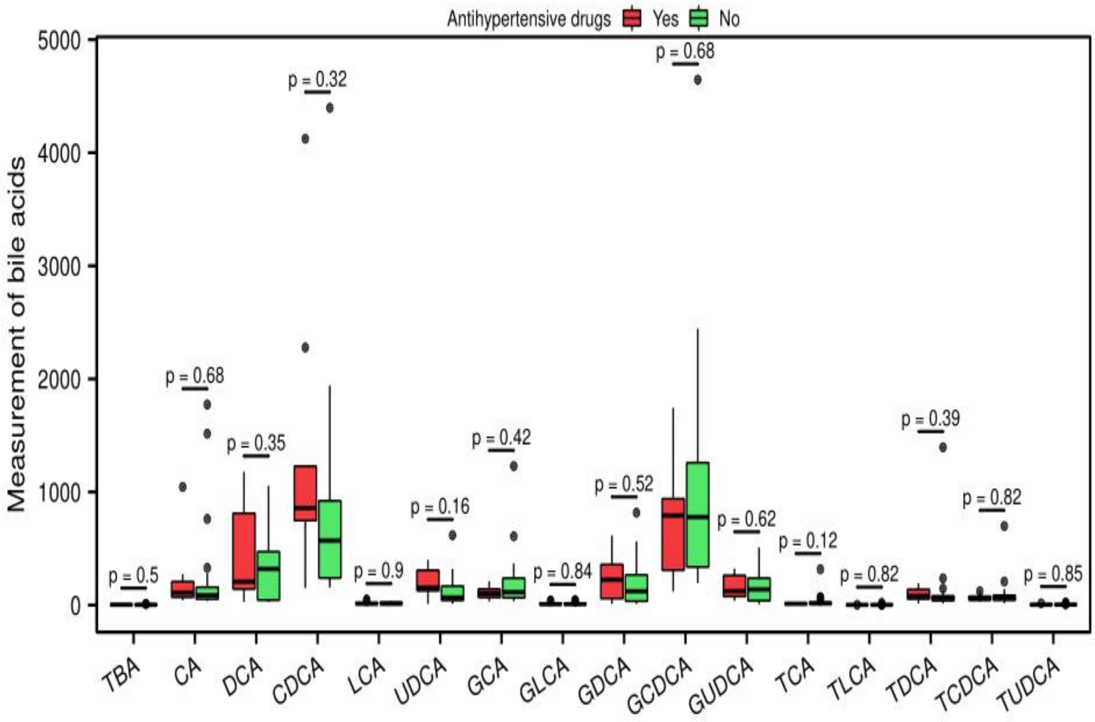

Supplement Figure 1C

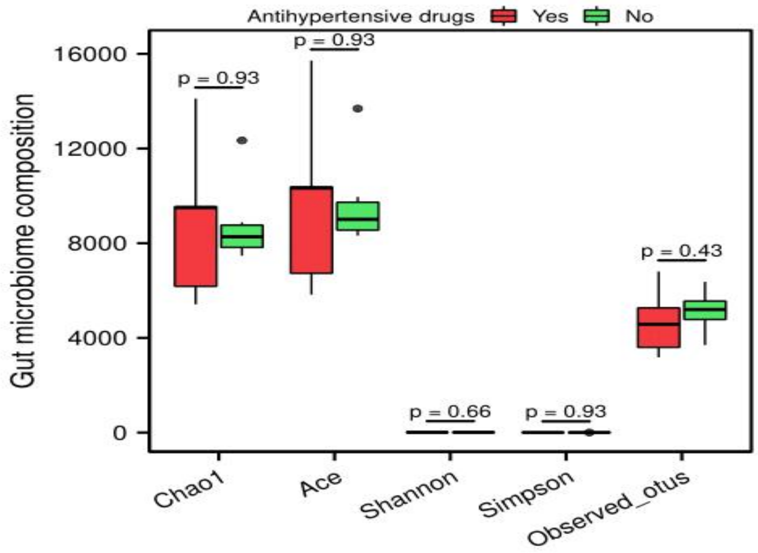

Supplement Figure 1D

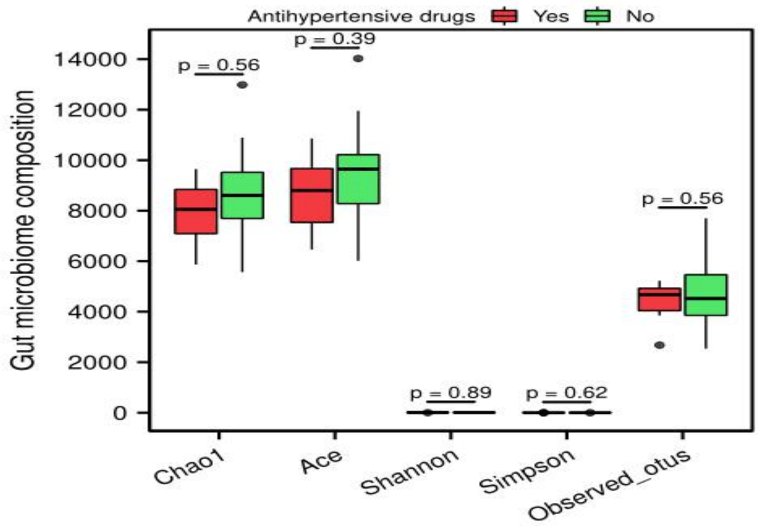

Supplement Figure 1: The Comparison of Bile Acids and Gut Microbiome Composition in Patients with and without Taking Antihypertensive medicines.

(A) The comparison of bile acids in H-CKD patients; (B) The comparison of bile acids in hypertension subjects; (C) The comparison of gut microbiome composition in H-CKD individuals; (D) The comparison of gut microbiome composition in hypertension cases. H-CKD, hypertension-related chronic kidney disease. No

significance differences of bile acids and gut microbiome composition found in patients with/without taking antihypertensive medicines (all p>0.05) in the both hypertension and H-CKD groups.

- **Supplement Figure 2**

Supplement Figure 2A

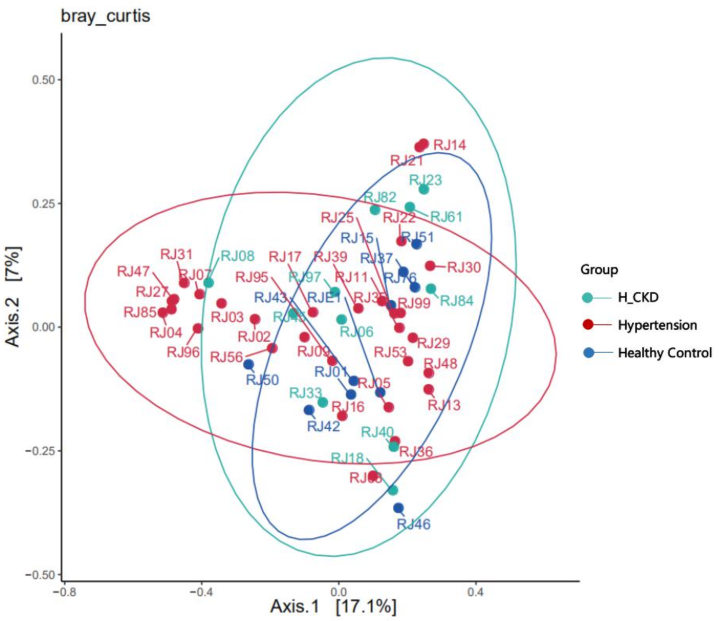

Supplement Figure 2B

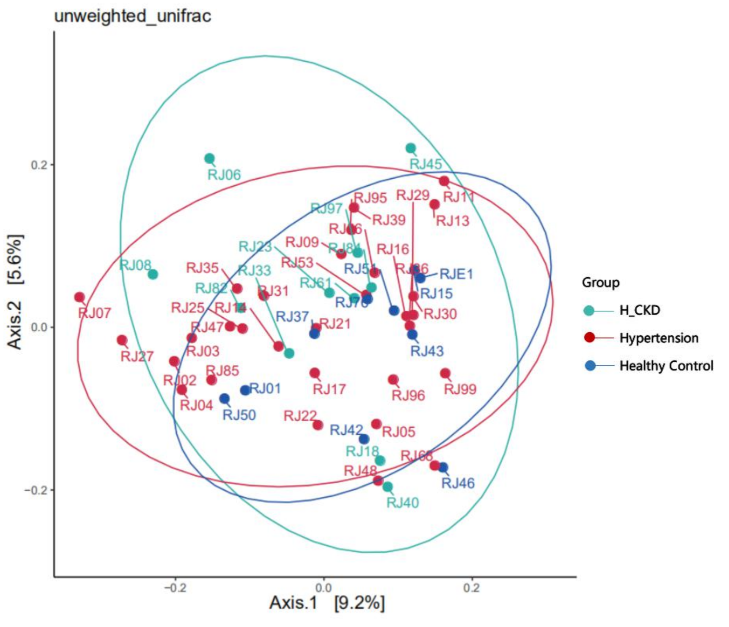

Supplement Figure 2: PCoA Analysis of Gut Bacteria among HC, H-CKD, Hypertension.

(A) Based on the Bray-curtis distance; (B) Based on the unweighted UniFrac distance. Different points or patterns represented different samples, each circle representing a group. The distance between the points or patterns is on behalf of the microbial structure difference.

- **Supplement Figure 3**

Supplement Figure 3A

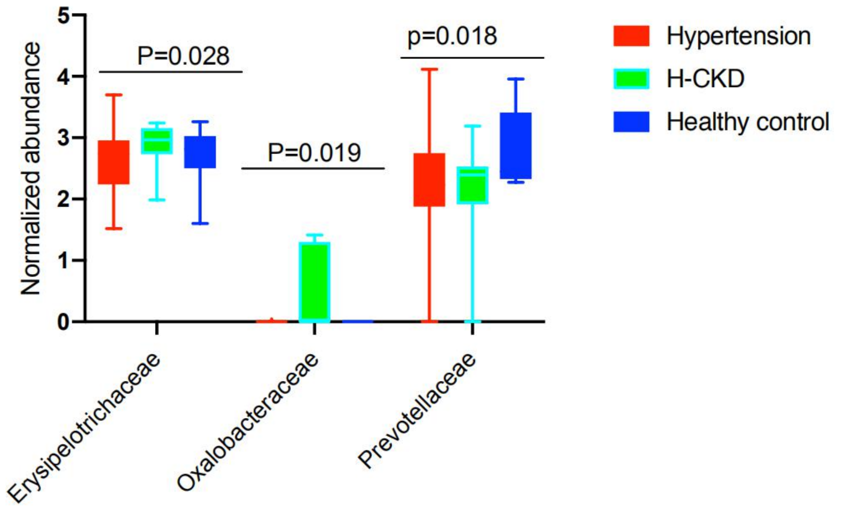

Supplement Figure 3B

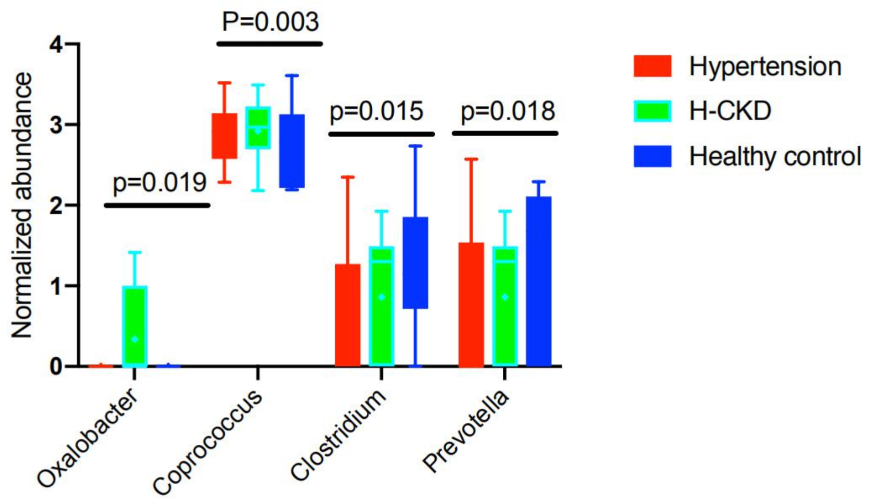

Supplement Figure 3C

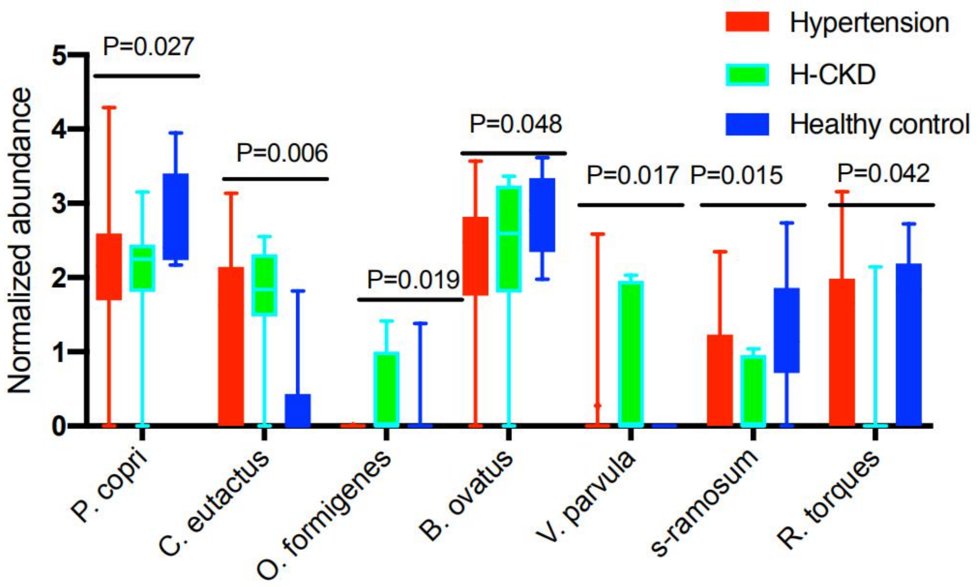

Supplement Figure 3: Normalized Abundance of Feature Bacteria in H-CKD, Hypertension and HC.

(A) Normalized abundance of feature bacteria in H-CKD, hypertension and healthy control at family level; (B) Normalized abundance of feature bacteria in H-CKD, hypertension and healthy control at genus level; (C) Normalized abundance of feature bacteria in H-CKD, hypertension and healthy control at species level. Points represent different samples; Normalized abundance: Log.10 transfer; P value calculated by Kruskal-Wallis test.
